# Book Reviews

**Published:** 1803-09-01

**Authors:** 


### CRITICAL ANALYSIS

OF THE

### RECENT PUBLICATIONS

ON THE

### DIFFERENT BRANCHES OF PHYSIC, SURGERY, AND MEDICAL PHILOSOPHY.

---

*The Edinburgh Practice of Physic, Surgery, and Midwifery, continued.* Vol. 3 and 4, Surgery. Vol. 5, Midwifery.

Two volumes of this very respectable Compilation are devoted to Surgery, a proportion of the whole adequate to the importance of the subject. The Editor has executed his task with the same industry and fidelity as in the former volumes, and, indeed, we think this part of the work in some respects superior to the former, a greater variety of authorities having been consulted, and the selection being more extensive. This indeed may be owing to the circumstance that no author in Surgery takes so decided a lead, and is resorted to with so preponderating authority as Cullen still retains in Physic. This obliges the Compiler to take a more extensive range, and enables him to enrich his work with much valuable matter that might have been lost or neglected if he had had before him a single compleat system, which the public had been in the habit of considering as standard authority. Still we must remark that the books which the Editor has consulted are all of easy access, almost all in the English language, and such as have a place in the commonest library. A quotation from Mr. Hunter, is followed by another from Mr. Bell, to which succeeds a page from Mr. Latta, and a communication from one of the valued Correspondents of our

Journal. In scarcely a single instance has the Editor thought proper to abbreviate and condense, still less has he indulged himself in comparing opinions, approving, or condemning.

We observe with pleasure, that those parts of Surgery which are of the most general use, and concern, more or less, every practitioner, are described with particular amplitude. Of this kind is the chapter on ulcers, burns, and the lues venerea. We shall not enumerate the leading contents of these volumes; suffice it to say, that every species of injury or disease requiring the assistance of the surgeon is noticed with considerable minuteness, nor have we discovered any omission of material importance in the execution.

The appendix contains a chapter on the important art of recovering suspended animation after drowning, and the plain, intelligible directions circulated by the Humane Society, are added with due commendation. It would have been of advantage if some notice had been taken of the other species of asphixia, as that from hanging, and from noxious vapours. The medical application of electricity, and the more recent discovery of Galvanism, also make a part of the appendix. Seven plates, exhibiting different kinds of surgical instruments and apparatus, are given at the end of the volume. They are executed in a very respectable style.

Vol. 5. *Midwifery.* The style of execution of this volume resembles that of the preceding; but, we think, not with so much propriety of selection. High as Dr. Smellie's authority stands, it can hardly be considered as superseding every other; but the work of this eminent accoucheur is here quoted with such profuse liberality, that the work of the scissars in a great degree supersedes that of the pen. This observation however only applies to a part of the volume, the remainder is given with more selection.

The chapter on Hysterotomy contains the unfortunate and much disputed Manchester case, but the Compiler appears in the history of this operation to be ignorant of the supposed extra-uterine cases, and of the general state of opinion on this subject. We do not even meet with the name of Dr. Osborne, nor any notice of his valuable observations on some important points of Midwifery.

The chapter on the diseases of children concludes with a short sketch of Vaccination by Mr. Ring.

Thirteen plates are added to illustrate the contents of this volume.

As the present work is a library in itself, (limited indeed, but containing little that will not prove of utility to the practitioner) the Editor has with great propriety given a copious index, a reference to the cases, a posological table, and other helps to the reader, the advantage of which every one in the habit of consulting books must be fully sensible of, and cannot easily overrate.

---

Observations

*Observations on Diarrhœa and Dysentery, as those Diseases appeared in the British Army during the Campaign in Egypt, in* 1801*; to which are prefixed a Description of the Climate of Egypt, and a Sketch of the Medical History of the Campaign. By* Henry Dewar, *late Assistant Surgeon to the Thirtieth, or Cambridgeshire Regiment of Foot.* London, 1803, pp. 161, 8vo.

The late campaign in Egypt, and the circumstance of its having been the seat of European war for some years, have made us pretty well acquainted with the medical topography of this singularly interesting country. Whatever be the event of military operations between European armies, medical science is uniformly enriched by them; for which, without going beyond our own country for examples, we may instance the labours of Lind, Huxham, and Clegborn. The diseases of Egypt have something as peculiar in their character, as the climate exhibits; the bilious, remittent, and destructive fevers of the tropical countries with which we are the best acquainted, appear to be much less frequent in the equally sultry country watered by the Nile; but the formidable diseases of the eyes, and the more terrible scourge of the country, the plague, make a weighty balance against the African or West-Indian fevers. Diseases of the bowels, however, appear to be equally frequent and destructive to Europeans in both situations, and to require much of the attention of the medical resident. The principal physician to the French army of Egypt, Desgenettes, makes the same observation as to the liability to bowel complaints, and it proved to be often severe, harassing, and not unfrequently fatal.

The introduction of the work before us, is short and not very interesting; it is a very partial sketch of the medical history, as it gives only an account of the circumstances which appeared to predispose the troops to the complaints which are afterwards described more at large. The author begins with diarrhœa. Here we must observe, that as these diseases have been so often and so well described, and as these symptoms in Egypt were not attended with any peculiar circumstances, it is chiefly in the plan of treatment, either for cure or prevention, that we can meet with any thing particularly worthy of attention, though the whole does credit to the author's abilities and observation. On the much agitated question of fruit being a cause of diarrhœa in hot countries, the author makes the following pertinent observations:

"It is a common thing, in table conversation, to discuss the comparative merits of different fruits, as well as of other articles of diet; and to ask any medical man who is present, his opinion of their tendency on the constitution. But it is seldom that an answer can be given to such questions, that will admit of universal application. Prompt and decisive replies are either the offspring of conceited ignorance, or are thrown out carelessly to fill up conversation, and satisfy the company at the time, while we are sensible that the opinion, opinion we give has no weight in our serious practice. I have heard it on such an occasion confidently asserted, that the most abundant use of water-melons never can do harm. Many who have eaten them freely in warm climates for a number of years, are ready to attest their innocence, and therefore treat the fears of others with ridicule. But I have known several cases in which this fruit has produced the most distressing complaints of the bowels. In these cases, a doubt could not exist of the cause of the complaint; because it commenced with a heavy sensation in the stomach, bearing a near resemblance to the taste of the fruit that had been eaten: a reluctance to this fruit was one attendant symptom of the disease; and if at any time, during convalescence, the patient was in the smallest degree betrayed into the farther use of water-melons, it was followed by a marked exacerbation of all his complaints.

"In the march to Cairo, when bowel complaints were most prevalent, the only fruits we had access to were cucumbers, melons, and apricots, with a few peaches. The apricots were often unripe, and sometimes they were soft and spoiled. The cucumbers and melons were seldom ripe, and were eaten by far too freely. These articles proved frequently exciting causes of diarrhœa. Their pernicious qualities might have been corrected by seasoning them with vinegar and pepper, particularly the latter. Every French soldier, I believe, kept a box of spices in his pocket. The same regulation among our troops would have more influence on the preservation of health in a warm climate, than such abundant supplies of animal food, without which an Englishman too often conceives himself starved."

The description of dysentery follows; and we may remark, that it is drawn up with great clearness, good sense, and accuracy. The author has indeed freely consulted Huxham and Sydenham, but he has, in several instances, very properly adapted the observations of these excellent writers to the circumstances of the Egyptian campaign, as in the following instance:

"It is necessary for Europeans, on removing to a hot climate, to employ a few simple precautions against this and other diseases. They ought, above all things, to avoid exposure to the immediate rays of the sun. They ought to conduct all their business through the day under a shade, and choose the cool of the evening, or rather the morning, after the fogs are dissipated, for walking abroad. If forced to go out at all in the middle of the day, they ought to use a very thick umbrella, forming a complete shade. The covering of the head should be of some thick white substance, and the European black hat should be laid aside. The white turban of the Turks is excellently adapted to a hot climate. Its white colour absorbs less of the heat of the sun than any other, and its numerous folds prevent the heat absorbed from penetrating to the head. In this country, indeed, such a covering would prove unpleasantly warm, as it would confine round the head the heat generated by the animal process. Here were find the external air agreeably cool, and feel rather rather oppressed, when much loaded with clothes on any part of the body. But, in such a climate as that of Egypt, the external heat so much exceeds any that is generated by the body itself, that a greater coolness is sometimes preserved, by confining the heat of our own bodies, than by exposing them to the external air. Those Oriental travellers, who exchange their hat for the turban, experience it to be a much cooler and more agreeable covering. The Turks and Egyptians, besides, keep their heads comfortable by constant shaving, and washing with cold water. Strangers have much greater occasion than the natives, for using such precautions. If they do not, the intense heat of the sun, oppressing the head, produces a debility which affects the whole body, and renders it liable to many other diseases as well as dysentery."

In the cure of this formidable disease, the author appears to lay much stress on flannel bandages applied to the abdomen. The advantage of this practice has often been experienced; and few practitioners have appeared to rely more on it than the author. As we chiefly wish to present to our readers what bears the sanction of actual experience, we shall extract some of the author's remarks.

"Warmth is not a secondary object. It is the very first that should be thought of. I shall give a particular account of what I reckon the best mode of covering the patient, and the mode which I followed in Egypt.

"Four or five folds of fine flannel, or a large piece of thick fleecy hosiery, ought to be laid over the abdomen, and, over this, a flannel bandage should be bound, rather tight, and in a uniform manner, from the groin, nearly to the arm-pits and back again. This mode of applying, or rather of confining, a certain degree of heat over that part of the body which is the seat of disease, is to be persisted in as long as the disease continues. When begun early, and well attended to, not neglecting the usual collateral means, it seldom fails to effect a cure. In whatever stage it is begun, with the exception of the very last, it produces a speedy amelioration of the symptoms, and cures many dysenteries that would otherwise be hopeless.

"The immediate effects of this swathing are, 1. The removal of that local torpor of the abdomen, under which a dysenteric patient often labours. Before its application, he feels as if he had no bowels, but when it is applied, the pressure which it makes restores over the whole abdomen those sensations which were deficient. 2. It obviates rawness and griping. Before applying it, the torpor which exists in the bowels is only interrupted by occasional gnawing sensations, which, on going off, leave him more torpid than before. The flannel bandage, by preventing those impressions of cold which form the chief cause of this uneasiness, does not fail to correct it. 3. It removes dejection and languor; the patient soon feels himself invigorated, and better fitted to relish the enjoyments of life. 4. It corrects that dyspnœa which is so often the consequence consequence of dysenteric debility. The support which it gives to the action of the abdominal muscles, and consequently of the diaphragm, enables the patient to respire with much less fatigue. This effect is experienced on the first application of the bandage; but if very tight, it will in a little time create a different sort of dyspnœa, of a less languid but more stifling nature, by preventing the full expansion of the lungs. In this case it must be made a little easier. The ultimate good effects of the flannel bandage are, an increase of general strength, and a healing process in the intestines, proceeding from an improved state of the sensation in those organs. Its good effects ought to be experienced very soon after it is applied. If, in two or three days it should produce no change, we must conclude, either that the symptoms proceed from such a fixed state of disease as requires other powerful remedies, or that the disease differs from the greater part of chronic dysenteries, which certainly owe their continuance and progress in a great measure to hurtful alternations of temperature.

" When the bandage is first applied, much of its efficacy arises from its pressure. Pressure, even with the palm of the hand, often gives a temporary relief to the bowels. But after it has been continued for a week or a fortnight, the pressure is sometimes of less service, and may be diminished. In some old dysenteries the bowels are so tender, that little or no pressure can be borne. But the warmth produced by the fur or fleecy wool under the bandage, is of more lasting benefit, and should be kept up without intermission till the dysentery is cured. Though the bandage should be applied very lightly over them, the elasticity of the soft wool makes them adhere so close as to exclude all cold, in a manner that cannot be effected by the mere use of a flannel shirt.

" In order to secure the good effects of the bandage, care must be taken that it be properly applied. In serious cases, I made a point of applying it myself, trusting most to its efficacy when I thus determined the exact degree of pressure that was made. Sometimes it was apt to loosen, and especially to move upward, so as to uncover the lower part of the abdomen. To prevent this, I first made it firm round one of the thighs, and after putting it once or twice about the body, brought it round to the opposite thigh, proceeding afterwards to apply it fully round the body. It was often necessary, especially when I was obliged to employ old bandages, to keep them together with pins in convenient places, after they were applied. But the bandages used, ought, if possible, to be of new flannel, for, after they are some time wore, they lose a great part of their elasticity. With these precautions, I always found them stay on as well as I could wish. But in private practice, where we meet with some corpulent subjects, this will often be more difficult. In such cases, an elastic jacket made of strong flannel, lined with soft wool over the abdomen, and fitted with elastic wires across the back, might answer all the purposes of the bandage. It should also have a strap fixed to the front part, to pass pass betwixt the thighs, and button again at the small of the back, in order to keep the jacket well down over the abdomen, and to protect the lower end of the rectum from cold, where that part is affected with morbid sensibility. The patent fleecy hosiery jackets sold in the shops, have not in general a thickness of wool on the part covering the abdomen, sufficient to answer the purpose; they are also too easy. Though elastic enough to apply closely to the body, I suspect they would not produce that degree of pressure on the abdomen which proves so useful, when coverings are first put on. When a jacket of this kind is to be used, an additional quantity of wool should be sewed to the inner part of the front, and the same kind of strap that I have mentioned, employed to keep it down over the belly. However, as this jacket produces but little sensible pressure, I should not altogether trust to it, without applying a roller over it, which might be pinned to the jacket, to prevent it from shifting.

This species of covering, in whatever form it is applied, ought to be kept on, even when the symptoms of dysentery begin to disappear; and, after they are gone, it ought to be laid aside with caution, and by slow degrees."

On the whole, we may consider this treatise as another instance of the excellent field of observation which the military hospital service furnishes, to those who have been rendered adequate to it by previous study.

